# Inter-Individual and Intra-Individual Relationships Between Neuroticism and Cognition: A Coordinated Analysis

**DOI:** 10.1093/geroni/igaa057.1248

**Published:** 2020-12-16

**Authors:** Tomiko Yoneda, Jonathan Rush, Graciela Muniz Terrera, Almar Kok, Boo Johansson, Scott Hofer, Daniel Mroczek, Andrea Piccinin

**Affiliations:** 1 University of Victoria, Victoria, British Columbia, Canada; 2 University of Edinburgh, University of Edinburgh, Scotland, United Kingdom; 3 Amsterdam University Medical Centres, Amsterdam, Netherlands; 4 University of Gothenburg, Göteborg, Sweden; 5 Northwestern University, Chicago, Illinois, United States; 6 Psychology, Victoria, British Columbia, Canada

## Abstract

Existing literature indicates a relatively consistent relationship between neuroticism and cognitive functioning (CF). Interindividually, high levels of neuroticism may predispose individuals to cognitive aging and dementia-related neuropathology. Intraindividually, increases in neuroticism may be intrinsic to the aging process or to dementia pathology. These hypotheses are not mutually exclusive, though the relationships are rarely examined using the same individuals, which may contribute to publication bias and confusion regarding the hypotheses as mutually exclusive. Data were drawn from the Origins of Variance in the Oldest-Old (Sweden, Mage=83.6, 67% female), Swedish Adoption/Twin Study of Aging (Sweden, Mage=60.4, 59% female), and Longitudinal Aging Study Amsterdam (Netherlands; Mage=68.1, 52% female). Controlling for age, sex, education, and depressive symptoms, parallel process latent growth models were fit independently in each sample (NT=3293) to simultaneously estimate growth parameters of neuroticism with three measures of CF (processing speed, learning/memory, and reasoning). Multilevel meta-analysis estimated the pooled covariation between neuroticism and CF at baseline and overtime, revealing a significantly negative intercept-intercept relationship across datasets (covariance= -0.46, 95% CIs [-0.90,-0.02], z=-2.02, p=0.04, τ2=0.06). The slope-slope covariances were consistently negative, but the meta-analytic pooled estimate was not significant despite some significant individual estimates across studies. Overall, results provide some evidence for intraindividual and interindividual relationships between neuroticism and CF, such that higher neuroticism is associated with lower CF, and neuroticism tends to increase as CF decreases. Identification of the early indicators and risk factors for cognitive decline may facilitate development of screening assessments and aid in treatment strategies for dementia care services.

